# Renal papillary tip extract stimulates BNP production and excretion from cardiomyocytes

**DOI:** 10.1371/journal.pone.0197078

**Published:** 2018-05-07

**Authors:** Itaru Goto, Ryuji Okamoto, Ryotaro Hashizume, Noboru Suzuki, Rie Ito, Keiichi Yamanaka, Hiromitsu Saito, Hiroshi Kiyonari, Isao Tawara, Yuki Kageyama, Yoshito Ogihara, Yusuf Ali, Norikazu Yamada, Naoyuki Katayama, Masaaki Ito

**Affiliations:** 1 Department of Cardiology and Nephrology, Mie University Graduate School of Medicine, Tsu, Mie, Japan; 2 Department of Pathology and Matrix Biology, Mie University Graduate School of Medicine, Tsu, Mie, Japan; 3 Department of Animal Genomics, Functional Genomics Institute, Mie University Life Science Research Center, Tsu, Mie, Japan; 4 Department of Dermatology, Mie University Graduate School of Medicine, Tsu, Mie, Japan; 5 Animal Resource Development Unit, RIKEN Center for Life Science Technologies, Kobe, Hyogo, Japan; 6 Genetic Engineering Team, RIKEN Center for Life Science Technologies, Kobe, Hyogo, Japan; 7 Department of Hematology and Oncology, Mie University Graduate School of Medicine, Tsu, Mie, Japan; University Medical Center Utrecht, NETHERLANDS

## Abstract

**Background:**

Brain natriuretic peptide (BNP) is an important biomarker for patients with cardiovascular diseases, including heart failure, hypertension and cardiac hypertrophy. It is also known that BNP levels are relatively higher in patients with chronic kidney disease and no heart disease; however, the mechanism remains unclear.

**Methods and results:**

We developed a BNP reporter mouse and occasionally found that this promoter was activated specifically in the papillary tip of the kidneys, and its activation was not accompanied by *BNP* mRNA expression. No evidence was found to support the existence of BNP isoforms or other nucleotide expression apart from BNP and tdTomato. The pBNP-tdTomato-positive cells were interstitial cells and were not proliferative. Unexpectedly, both the expression and secretion of BNP increased in primary cultured neonatal cardiomyocytes after their treatment with an extract of the renal papillary tip. Intraperitoneal injection of the extract of the papillary tips reduced blood pressure from 210 mmHg to 165 mmHg, the decrease being accompanied by an increase in serum BNP and urinary cGMP production in stroke-prone spontaneously hypertensive (SHR-SP) rats. Furthermore the induction of BNP by the papillary extract from rats with heart failure due to myocardial infarction was increased in cardiomyocytes.

**Conclusions:**

These results suggested that the papillary tip express a substance that can stimulate BNP production and secretion from cardiomyocytes.

## Introduction

Brain natriuretic peptide (BNP) belongs to a family of vasoactive peptide hormones with favorable physiological properties [1]. BNP is an established diagnostic biomarker for patients with cardiovascular diseases, including heart failure [2] and cardiac hypertensive hypertrophy [3]. The biological effects of BNP include vasorelaxation, natriuresis and diuresis, leading to the regulation of blood pressure and body fluid volume [4]. In humans, hypertension is a characteristic of individuals with deficiencies in the natriuretic peptide system [5, 6], while those who genetically have a higher basal concentration of BNP have lower systolic and diastolic blood pressure [7]. However, it remains unknown how BNP is regulated, especially in terms of its upstream signaling. It is believed that a stretch receptor, which has not yet been identified correctly, can stimulate the expression of BNP in proportion to ventricular wall stress [8]. Additionally, it is known that BNP levels are relatively higher in women, patients with chronic kidney disease and in elderly people with no heart disease [9]. We hypothesized that the renal papillary tip plays an important role in regulating the expression of BNP and our aim to determine whether a substance in the renal papillary tip is associated with BNP or not.

## Materials and methods

### Animal preparation

All protocols were approved by the Animal Care and Use Committees of Mie University (protocol No. 24–25) and RIKEN Kobe Branch (AH13-03).

#### Development of the pBNP-tdTomato Tg mice

**Fig 1:**
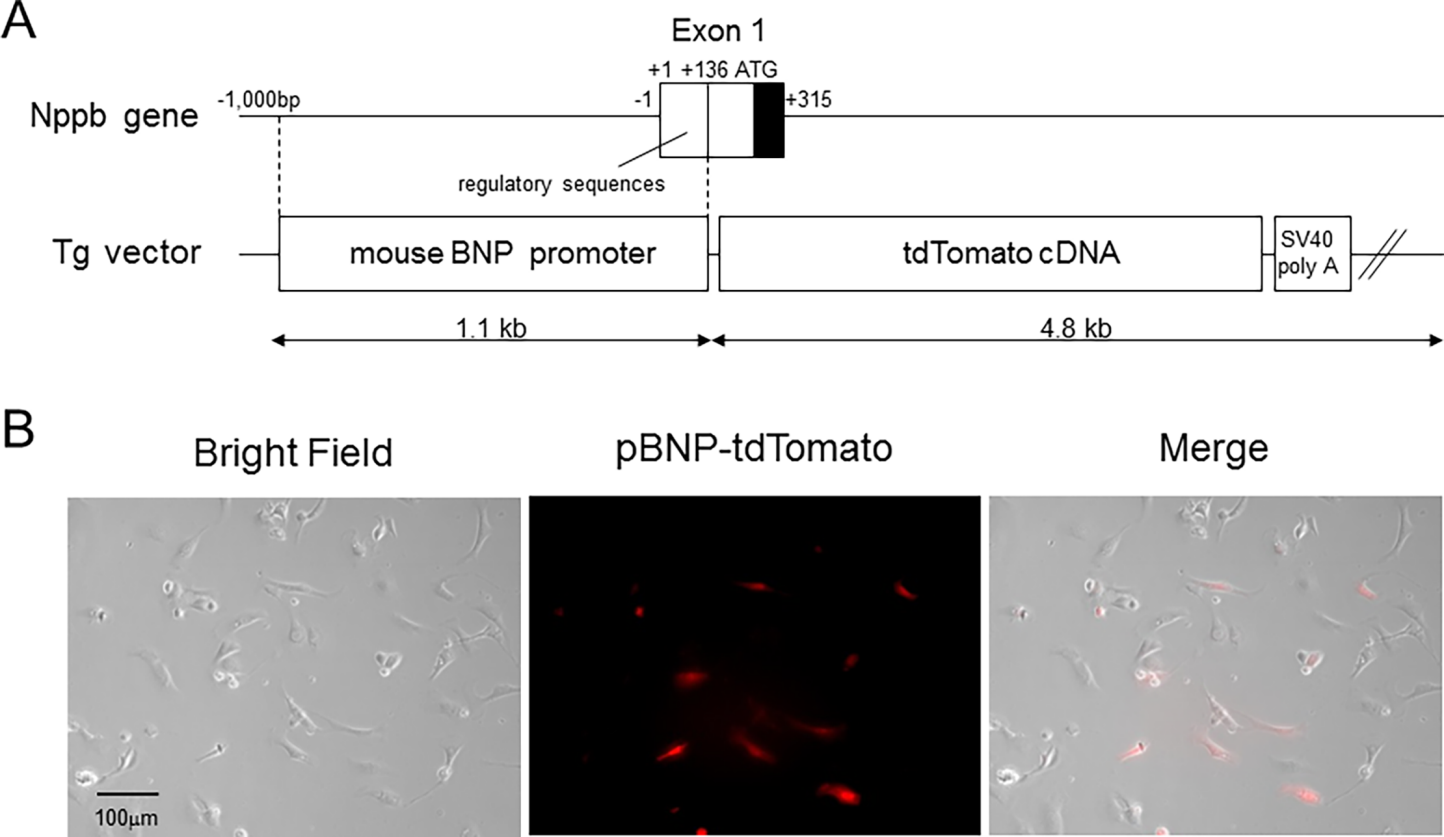
tdTomato was expressed specifically in cardiomyocytes from pBNP-tdTomato transgenic mice. A, Transgenic (Tg) vector of pBNP-tdTomato. B, Isolated cardiomyocytes expressing tdTomato and mixed fibroblasts with no expression of tdTomato in a BNP reporter mouse. The pictures are representative of five mice.

A 1136-bp fragment of the mouse *NPPB* gene 5’ flanking region (GenBank #NC_000070.6) from -1000 to +136 was amplified from C57BL/6 mouse genomic DNA by the polymerase chain reaction (PCR) technique using KOD Plus™ DNA polymerase (Toyobo, Osaka, Japan) and the following primers: 5’-GCGGATCCCCTGGTCATTGTCCTTGACCAACCT-3’ and 5’-ACGGATCCCTCCCCAAGCAGCTTGTGCACTGG-3’, which introduce *BamH*I sites (underlined) into both the 5’ and 3’ ends. The PCR products were subcloned into the *BamH*I site of the promoterless ptdTomato vector. Each fragment was sequenced to confirm that the PCR-amplified cDNA was identical to the original sequence. The promoterless ptdTomato vector was constructed by removing the ZsGreen cDNA from the promoterless pZsGreen 1.1 vector (Takara, Kusatsu, Japan) by digestion with *BamH*I + *Not*I, and replacing it with the tdTomato cDNA fragment from the ptdTomato N1 vector (Takara), which was also digested with *BamH*I + *Not*I. This transgene (Fig 1A) was used for pronuclear microinjection into fertilized C57BL/6N mouse eggs at the RIKEN Center for Developmental Biology (CDB). The Tg mice were identified by PCR assay using genomic DNA extracted from tail tissue. Six independent founder lines were identified and crossed with C57BL/6J wild-type mice to generate wild-type and transgenic offspring with a pure C57BL/6J genetic background. Line 6 (Tg(L6)) showed high expression levels of tdTomato on Western blotting analysis. Tg(L6) mice were mainly used between 4 weeks and 22 months of age. Tg(L2) mice showed modest expression levels of tdTomato. Tg(L6) is available (R. Okamoto or M. Ito: mitoka@clin.medic.mie-u.ac.jp; Accession. No. CDB0515T; http://www2.clst.riken.jp/arg/TG mutant mice list.html).

Rat renal papillary tips were prepared from 6-week-old male Sprague-Dawley (SD) rats and 10 to 12-week-old female Lewis rats (Japan SLC, Hamamatsu, Japan).

Stroke-prone spontaneously hypertensive male rats (SHR-SP, 10 to 11 weeks old) were obtained from the Disease Model Cooperative Research Association (Kyoto, Japan). Systolic blood pressure was measured using the tail-cuff method (Softron Co. Ltd., Tokyo, Japan).

### Cell and tissue culture

Mouse and rat neonatal cardiomyocytes were isolated from the ventricles of pBNP-tdTomato Tg mice and SD rats as described previously [10]. Cardiomyocytes were grown in growth media in a fibronectin-coated 12-well dish for 24 h.

Renal medullary interstitial cells were obtained from the fresh renal papillary tips of pBNP-tdTomato Tg mice, as previously reported [11]. Briefly, the papillary tip was dissected from the kidney under a stereoscopic microscope, minced with micro-scissors, and incubated for 75 min at 37°C in HEPES-Ringer’s buffer containing 0.2% collagenase type 2 (Invitrogen, Waltham, USA) and 0.2% hyaluronidase (Sigma, St. Louis, MO). Cells were further dissociated by gently passing the tissue through 23G and 27G needles. Dissociated cells were plated into Dulbecco’s modified Eagle’s medium (DMEM) containing 1 g/L glucose and supplemented with 10% fetal bovine serum, 50 U/L penicillin, 50 U/L streptomycin, 1% MEM non-essential amino acids, 1% L-glutamine and 1% sodium pyruvate (all from Invitrogen).

For tissue culture, the papillary tip was cut from the kidneys of pBNP-tdTomato mice and transferred onto a 35-mm dish with sterile forceps. A coverslip was placed onto the tissues and DMEM was added. The plate was incubated at 37°C in an atmosphere of 5% CO_2_ for 1 week. Subsequently, the medium was changed three times a week. Cells and cultured tissues were observed using a fluorescent microscope (BZ-X710, Keyence, Osaka, Japan).

For real-time quantitative PCR, pBNP-tdTomato-positive cells were isolated based on tdTomato expression using a fluorescence activated cell sorting (FACS) Aria device (BD, Biosciences, San Jose, CA, USA) (S2 Fig) [12].

Incubation of rat neonatal cardiomyocytes with the extract of the papillary tip or the inner medulla was performed as follows. The papillary tip and renal inner medulla were dissected from eight kidneys under a stereoscopic microscope, minced with micro -scissors and homogenized in 200 μl of DMSO with a small teflon homogenizer, followed by the addition of 4 ml of extraction buffer A containing 30 mM Tris-HCl, pH 7.5, 0.3 M NaCl, 1 μM (p-amidinophenyl)methanesulfonyl fluoride (aPMSF) and a cocktail of proteinase inhibitors (Complete Mini, Roche, Basel, Switzerland). The homogenate was centrifuged at 15,000 × *g* for 10 min. After the supernatant was passed through 0.22 μm sterile filters, the protein concentration was measured, and equal amounts of protein (≈100 μg) were added to fresh medium and used to treat rat cardiomyocytes for 24 h.

### Histological analysis

Kidneys were perfused with phosphate-buffered saline followed by fixation with 4% paraformaldehyde. They were then embedded in Tissue-Tek OCT compound (Sakura Finetek, Tokyo, Japan) and snap-frozen in liquid nitrogen. Unstained cryosections (6 μm) were observed using a BZ-X710 fluorescent microscope and BZ-X Analyzer software (Keyence). Some cryosections were stained with hematoxylin/eosin for whole imaging.

### Northern blot analysis

Total RNA was isolated from the heart and the upper and lower poles and pelvis of the kidney, including the papillary regions, with TRIzol reagents (Invitrogen) according to the manufacturer’s protocol. Equal amounts of total RNA (20 μg) were separated by 1.0% formaldehyde agarose gel electrophoresis, and were then transferred to Hybond-N+ (GE, Chicago, IL), as described previously [13]. Northern blot probes of mouse BNP cDNA from the start codon to the stop codon were generated by PCR using the following primer set: 5’ probe, forward, 5’- ATGGATCTCCTGAAGGTGCT -3’; reverse, 5’- CTACAACAACTTCAGTGCGT -3’. The probe was labeled with a random primer DNA labeling kit (Takara). The membrane was hybridized to the ^32^P-labeled probe in Rapid-Hyb Buffer (GE) at 65°C for 2 h. The hybridized membrane was visualized using autoradiography.

### Real-time quantitative RT-PCR

Total RNA was extracted from tdTomato-positive cells with TRIzol. Real-time quantitative RT-PCR was performed on cDNA generated from total RNA using PrimeScript Reverse Transcriptase (Takara) and random hexamers, as described previously [14]. For PCR, we used Assay-on-Demand Gene Expression Products as the predesigned sense and antisense primers, TaqMan Gene Expression Assays (Applied Biosystems, Foster City, CA, USA) and an ABI 7300 Real-Time PCR System (Applied Biosystems). The TaqMan probes used included Fos Mm00487425_m1, Gata4 Mm00484689_m1, Tead1 Mm00493507_m1 and Gapdh Mm99999915_g1.

### Proteolysis and molecular-sized fractionation of extracts from rat renal papillary tips

#### Proteolysis of tip extracts with proteinase K

Renal papillary tip extract was digested at 25°C with 0.3 μg/ml proteinase K (Wako, Osaka, Japan) in 30 mM Tris-HCl, pH 7.5, 0.3 M NaCl. At the indicated time points, an aliquot was removed and the digestion was terminated with the addition of aPMSF (final to 1 μM) and a cocktail of proteinase inhibitors.

Tip extract protein samples were fractioned using 100, 50, 30 and 10 kDa molecular weight cut-off (MWCO) Amicon Ultra-4 centrifugal filter units (Millipore, Germany) at 1,500 x g, by diafiltration according to the manufacturer’s protocol.

### Intraperitoneal injection of the extract of the renal papillary tip into SHR-SP rats

Extracts of the papillary tip and renal medulla were prepared as described above. Equal amounts of protein (≈320 μg) were injected intraperitoneally into four SHR-SP rats. The rats were then placed individually in metabolic cages and urine samples were collected 24 h after the injection.

### Blood and urine measurements

Serum and urine electrolytes, urea nitrogen, creatinine and osmolality were measured by a clinical laboratory testing service (SRL, Tokyo, Japan). Enzyme-linked immunosorbent assay kits were used for evaluating the levels of BNP-45, a major form of rat BNP (AssayPro, St. Charles, MO), and of cGMP (Abcam, Cambridge, UK).

### Left anterior descending coronary artery ligation in Lewis rats

Myocardial infarction was studied in 10 to 12-week-old female Lewis rats, as described previously [15]. Anesthesia was induced with 3.0% isoflurane inhalation with 100% oxygen, followed by intubation and respiratory support with a rodent volume-controlled mechanical ventilator (VentElite 55–7040, Harvard Apparatus, Holliston, MA) at a tidal volume of 3 mL and 80 breaths/min. A 4^th^ left thoracotomy was performed to expose the heart, following which the proximal left anterior descending coronary artery was ligated with a 7–0 polypropylene suture. Myocardial ischemia was confirmed by decreased movement in the left ventricle free wall and regional cyanosis. Five days after induction of myocardial infarction, the rats were sacrificed and their blood and hearts were collected for serum and histological analysis.

### Statistics

Data are reported as the mean ± standard error of the mean and were compared using two-tailed Student’s *t* tests. Systolic blood pressure was analyzed by two-way ANOVA followed by post-hoc analysis of Tukey’s test. A value of P < 0.05 was considered to be statistically significant.

## Results

### Development of a BNP promoter reporter mouse and investigation of activation of the BNP promoter in aged female mice

**Fig 2:**
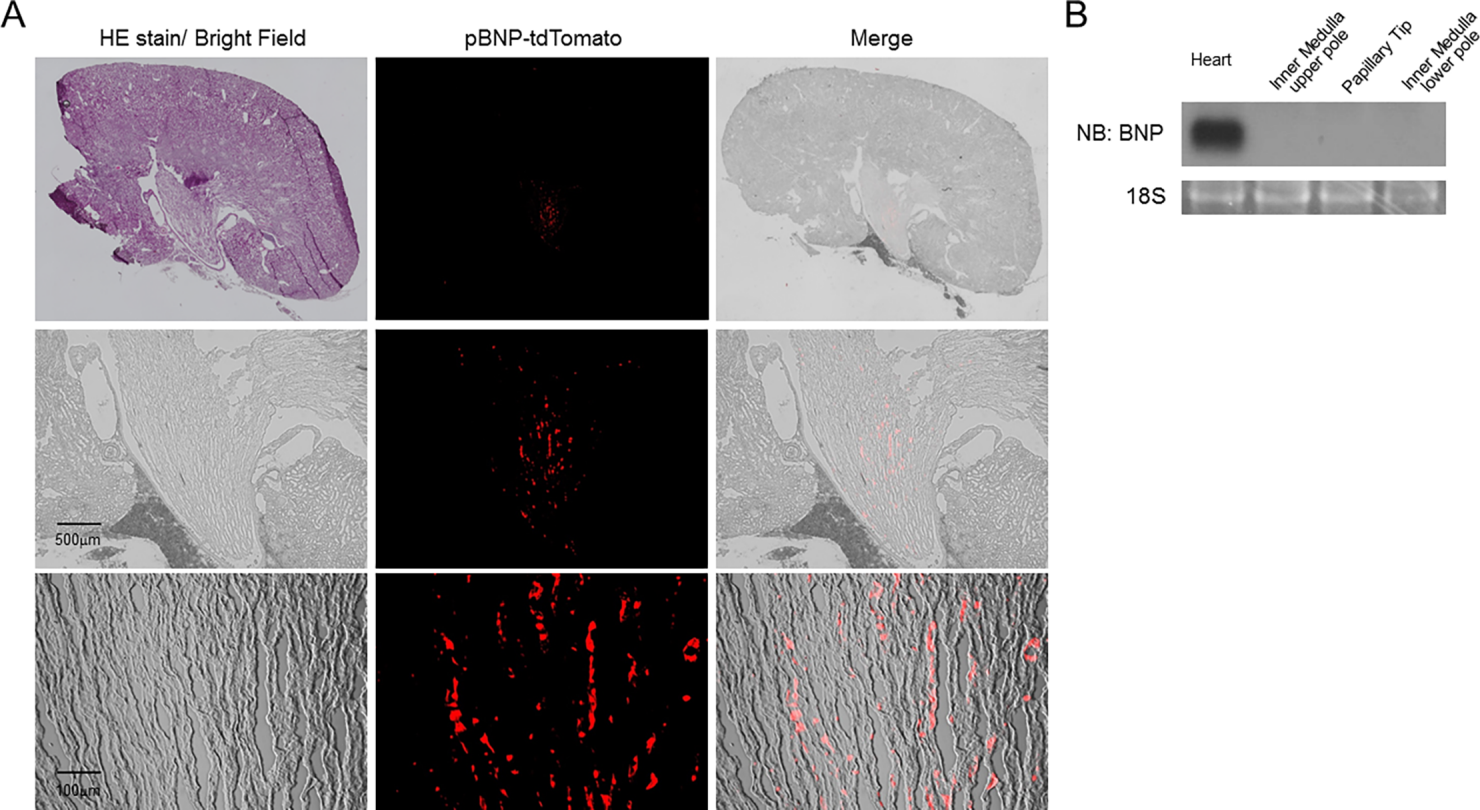
Activation of the BNP promoter in the papillary tip from the kidneys of pBNP-tdTomato Tg mice with no expression of *BNP* mRNA. A, Upper panels, whole imaging (bright-field) of a kidney stained with hematoxylin-eosin (HE), endogenous signal of tdTomato and merged images of frozen sections. Magnifications of the top panels are shown in the middle and lower panels. The pictures are representative of six mice. B, Northern blot analysis of BNP mRNA expression in the heart, inner medulla in the upper and lower poles and the papillary tip of the kidneys (n = 4).

**Fig 3:**
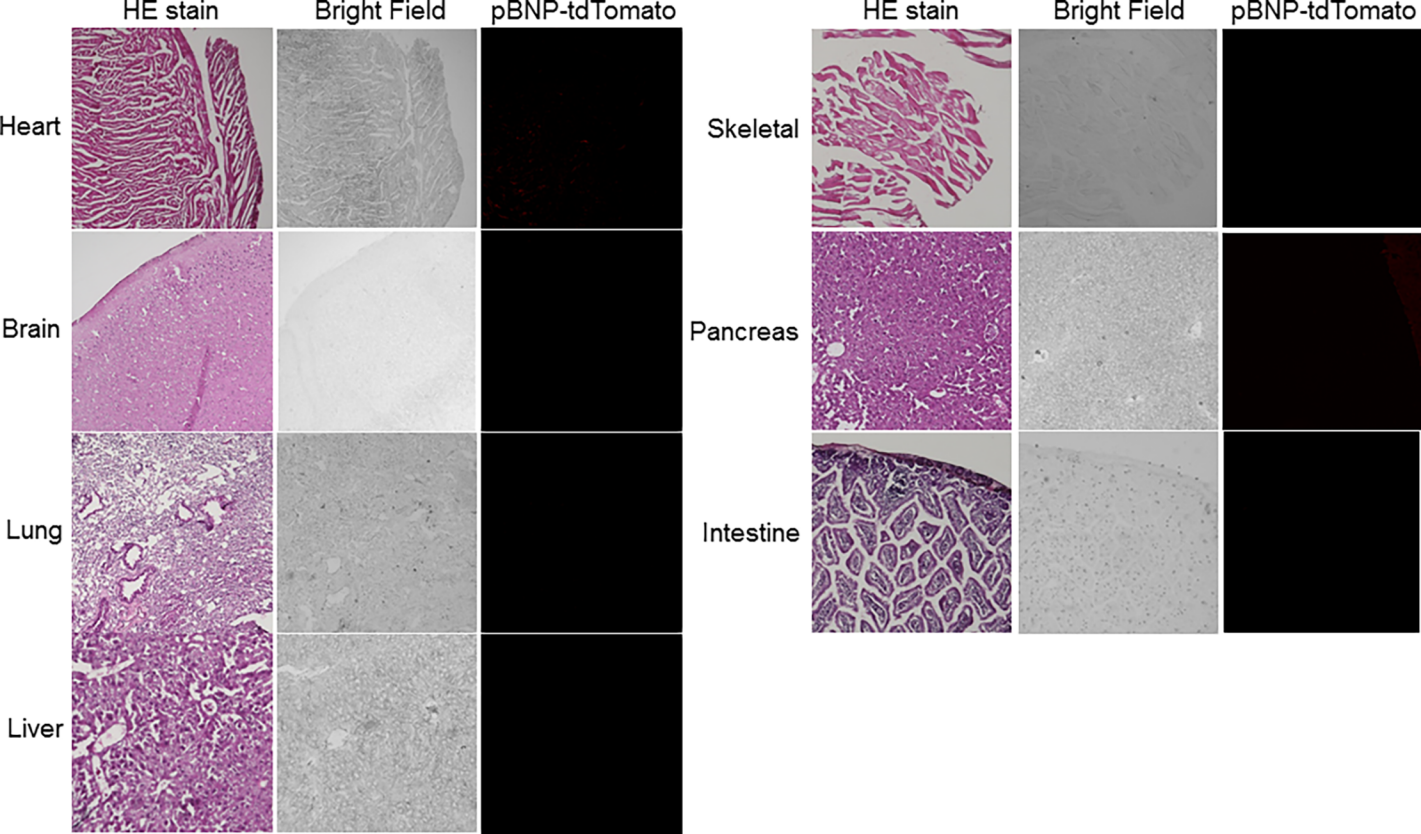
Mild activation of the BNP promoter in the heart, with no activation in the other organs, of pBNP-tdTomato Tg mice. The pictures are representative of three mice.

In this study, we generated transgenic (Tg) mouse lines carrying tdTomato under the promoter of the 1.1-kb mouse BNP gene fragment (Fig 1A), and investigated BNP promoter activation in different tissues of 22-month-old female mice by immunofluorescent analysis of tdTomato expression. In addition to immunofluorescent detection of the expression of tdTomato in neonatal cardiomyocytes (Fig 1B), we occasionally found a strong tdTomato signal in the papillary tip of the kidneys (Fig 2A). Similar results were obtained in young and/or male mice, although we could not observe any expression of tdTomato in the kidneys from 1-day-old pups (data not shown). The strongest tdTomato signal was observed in the papillary tips of the kidneys from all the adult mice (n > 20). pBNP-tdTomato-positive cells appeared to be interstitial and were not observed in any other area of the kidneys, except for the papillary tip (Fig 2 A). Interestingly, the signal of pBNP-tdTomato was more intense in the papillary tip than in any other organ, including the heart (Fig 3). To exclude possible artifacts due to overexposure to fluorescent light, we carefully observed the papillary tip under overexposure to green fluorescent light; we did not observe any green signal co-localizing with pBNP-tdTomato (data not shown). In addition, we did not observe any expression of *BNP* mRNA in the papillary tip or in the upper or lower renal inner medulla (Fig 2B). To examine whether BNP isoforms or other gene products, such as non-coding RNA, might be expressed from the transgene, we tried to amplify the transgene products using RT-PCR of mRNA from the papillary tip with specific primers to the BNP promoter region, Oligo(dT) or random primers. However, we could not amplify any gene products, except for tdTomato itself. 5’ Rapid amplification of cDNA ends (5’ RACE) did not amplify the 5’ end of the mRNA beyond a reported transcriptional initiation site of BNP. In situ hybridization of BNP, which could stain local upregulation of BNP in mice hearts three days after left coronary artery ligation surgery (S1A Fig), did not show any co-localization of ISH stain and pBNP-tdTomato fluorescence in Tg mice (S1B Fig).

### Effect of the extract from the papillary tips on the expression and excretion of BNP

**Fig 4:**
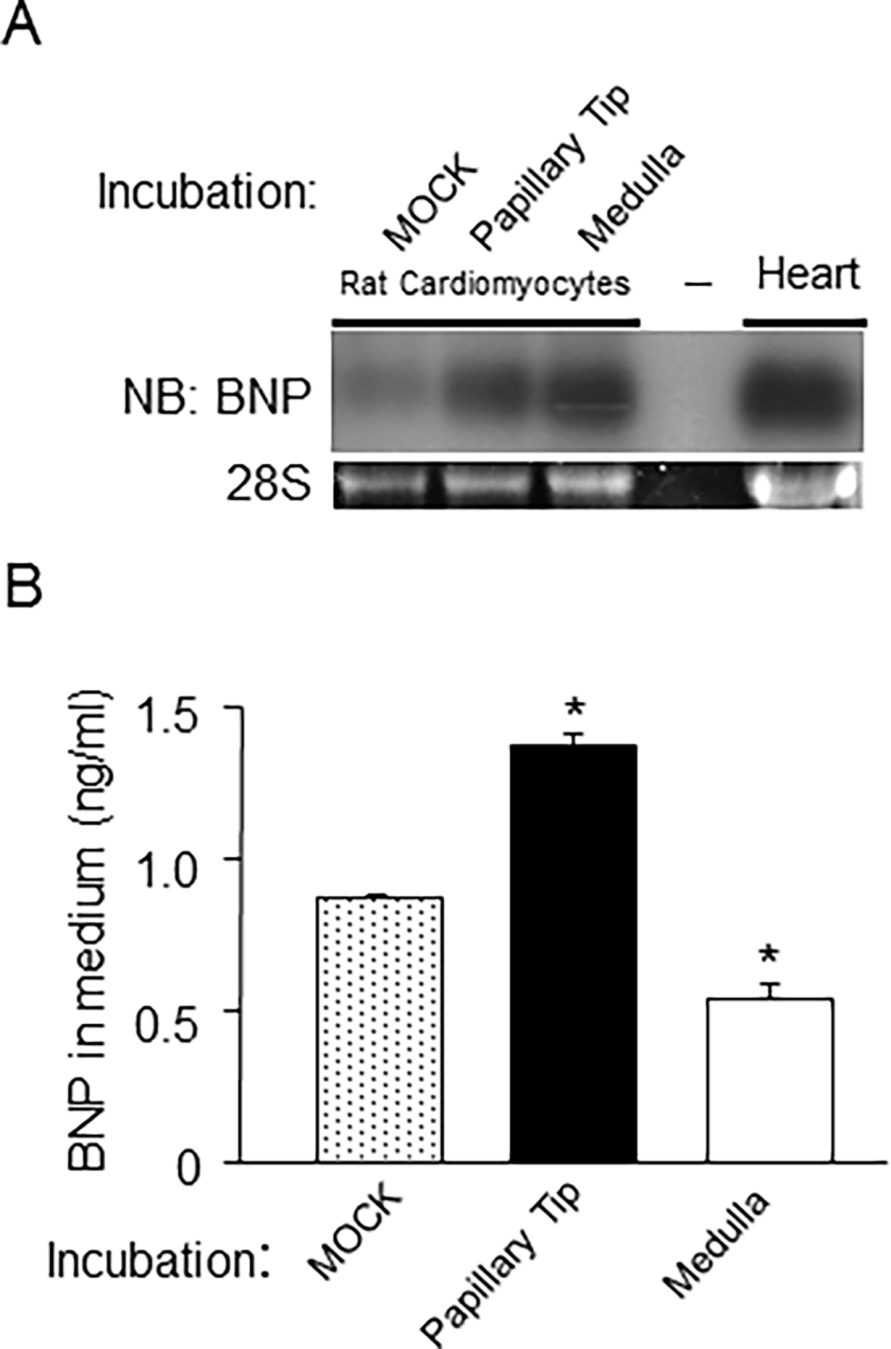
Treatment with an extract of the papillary tip of the kidneys increased the expression and secretion of BNP in rat neonatal cardiomyocytes. A, BNP mRNA expression was examined by Northern blot analysis in cardiomyocytes treated with control buffer or an extract of the papillary tip or the inner medulla of the kidneys. BNP expression in the heart was used as a positive control. B, Secreted BNP was examined using a rat BNP enzyme-linked immunosorbent assay kit. *P < 0.05 vs. medium from cardiomyocytes treated with the buffer alone (MOCK). n = 4. Data represent the mean +/- standard error of the mean.

We hypothesized that the papillary tip plays an important role in regulating the expression of BNP via an unknown mechanism, and, therefore, investigated the effects of an extract of the rat papillary tip on the expression of BNP in cultured rat neonatal cardiomyocytes. Surprisingly, since no BNP expression was detected in the presence of the transgene, mRNA expression of BNP by cardiomyocytes increased after a 24-h treatment with an extract of the renal papillary tip or inner medulla, while buffer alone had no effect on the expression of BNP by cardiomyocytes (Fig 4A). Furthermore, more BNP was secreted into the medium after treatment of the cardiomyocytes with the papillary tip extract than after treatment with the extract of the inner medulla or buffer alone (Fig 4B). Although treatment with the extract of the inner medulla increased BNP mRNA in the cardiomyocytes, it conversely inhibited BNP secretion by these cells. Similar results were obtained in young and/or male mice (data not shown). Evaluations to exclude artifacts due to contamination by angiotensin II, endothelin 1, and type A, B and C natriuretic peptides indicated no change in the expression of these peptides between the papillary tip and other portions of the kidneys (data not shown). These results suggested that the papillary tip extract could directly stimulate both the expression and excretion of BNP in cardiomyocytes.

### Characterization of proteins in the extract from the papillary tips

**Fig 5:**
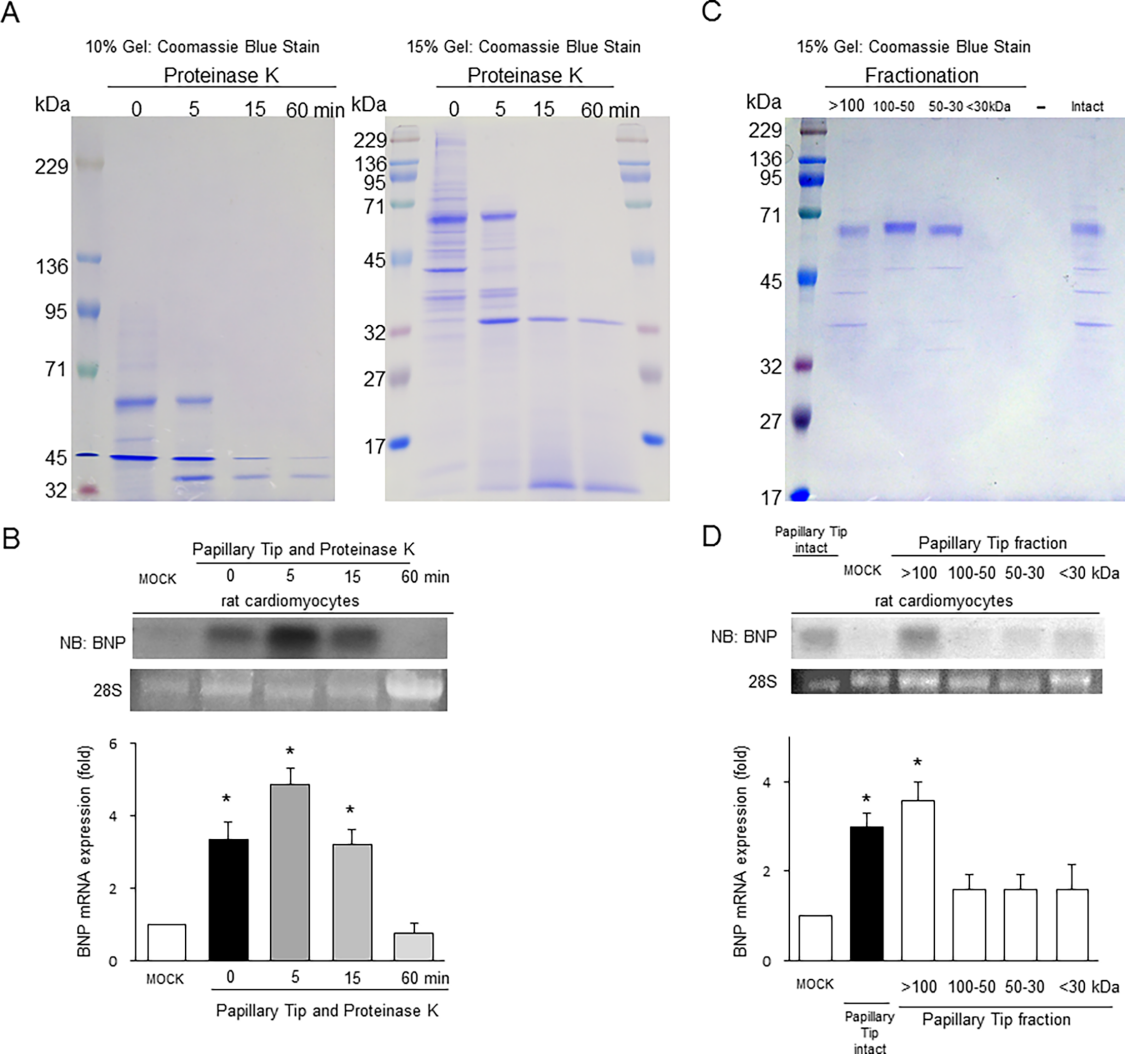
Characterization of papillary extracts by proteolysis and molecular weight fractionation. A, Papillary extracts were digested with 0.3 μg/ml Proteinase K for each indicated time and loaded into 10% and 15% gel. The pictures are representative of three experiments. B, BNP mRNA expression was examined by Northern blot analysis in cardiomyocytes treated with control buffer (MOCK) or each digested extract of the papillary tip. *P < 0.05 vs. cardiomyocytes treated with the buffer alone (MOCK). n = 4. C, Papillary extracts were fractionated according to their molecular weight range. The pictures are representative of three experiments. D, BNP mRNA expression was examined by Northern blot analysis in cardiomyocytes treated with control buffer or each molecular-weight-fractionated extract of the papillary tip. *P < 0.05 vs. cardiomyocytes treated with the buffer alone (MOCK). n = 3.

To determine the nature of the substance that stimulates BNP production, we initially treated extracts with proteinase K. Most of the proteins contained in the papillary extract had a MW of less than 71 kDa (Fig 5A). Treatment with 0.3 μg/ml proteinase K resulted in digestion of the extracts in 60 min (Fig 5A). We observed increases in BNP expression in rat neonatal cardiomyocytes after treatment with the extracts for 0 min, 5 min, 15 min and 60 min (Fig 5B), suggesting that this molecule is a protein and that limited proteolysis may stimulate the induction of BNP.

Next, we fractionated the extract with filter units for each molecular weight cut-off. Among them, a fraction (>100 kDa) was able to induce an increase in BNP expression in cultured cardiomyocytes.

### Characterization of pBNP-tdTomato-positive cells

**Fig 6:**
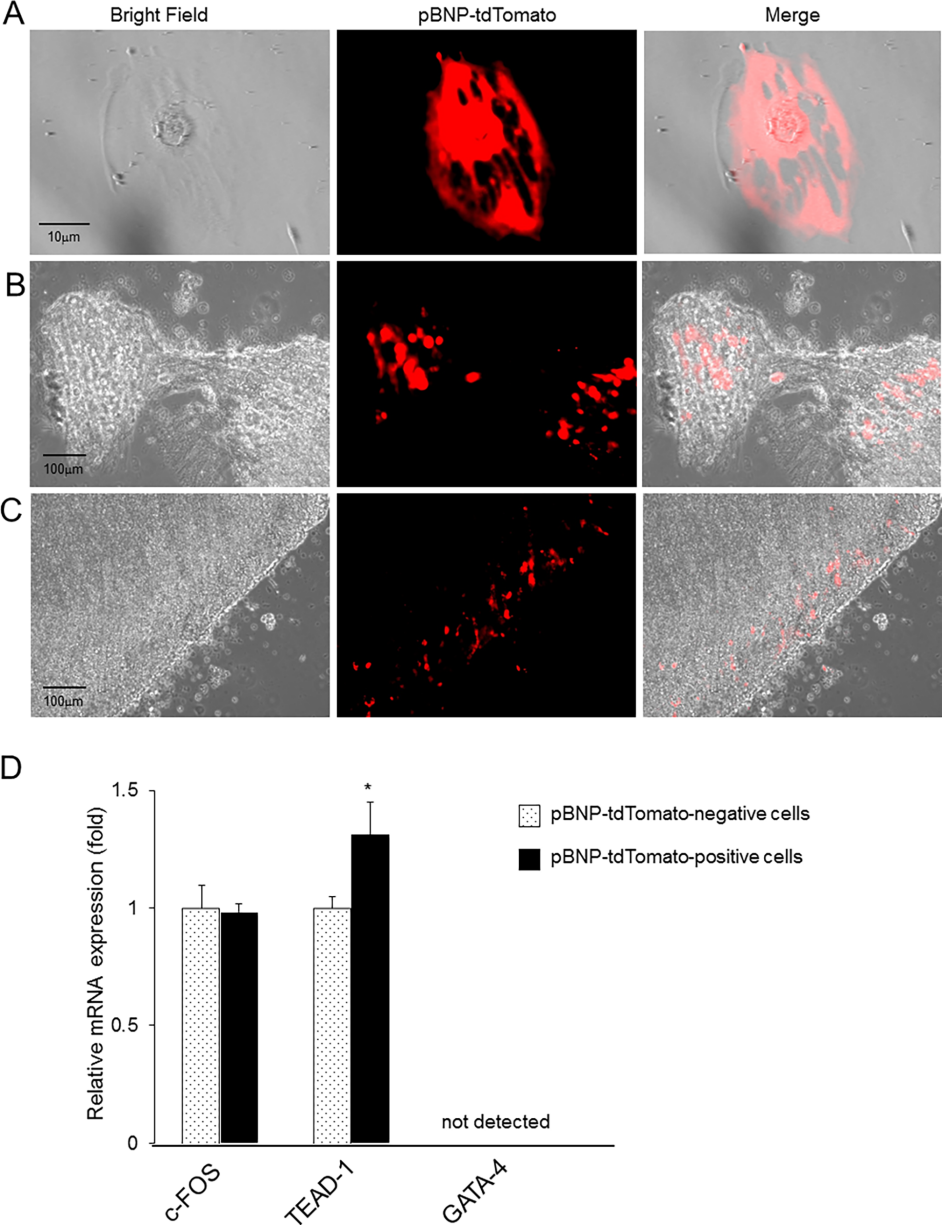
pBNP-tdTomato-positive cells were interstitial cells and were not proliferative. A, Endogenous tdTomato expression in an isolated renomedullary interstitial cell from a Tg mouse. The pictures are representative of five experiments. B, tdTomato expression in the cultured tissues of a papillary tip from a pBNP-tdTomato Tg mouse on day 6 and C, day 28. The pictures are representative of five mice. D, Real-time PCR showing c-FOS, GATA-4 and TEAD-1 expression in pBNP-tdTomato-positive and -negative cells isolated by fluorescence activated cell sorting (FACS) from renal papillary tips of pBNP-tdTomato Tg mice. *P < 0.05 vs. pBNP-tdTomato-negative cells. n = 4. Data indicate the mean +/- standard error of the mean.

Next, to characterize the pBNP-tdTomato-positive cells, we attempted to establish a primary culture of the cells, although cell viability was very low. The few viable cells obtained were cylindrical in shape and had round nuclei (Fig 6A). We cultured the papillary tip as a tissue under coverslips and found that the tdTomato-positive cells were interstitial cylindrical cells in the papillary tissue, similar to those observed in kidney sections (Figs 2A and 6B). Although we were able to culture papillary tip tissue containing tdTomato-positive cells for more than 2 months, no proliferation was observed. The tdTomato-positive cells were localized near the outline margins of the papillary tip tissue, but did not sprout out aggressively (Fig 6C). Since we failed to obtain and isolate sufficient amounts of viable and proliferative pBNP-tdTomato-positive cells, we isolated pBNP-tdTomato-positive and -negative cells using FACS from papillary tips from Tg mice (S2 Fig). We performed real-time PCR assay and analyzed the expression of transcription factors that play important roles in BNP expression [8], such as c-FOS, a subunit of activator protein-1 (AP-1), GATA-4 and TEA domain transcription factor-1 (TEAD-1). Real-time PCR showed an increase in TEAD-1, but not in c-FOS, in pBNP-tdTomato-positive cells compared with pBNP-tdTomato-negative cells (Fig 6D). GATA-4 could not be amplified in both cells. Thus, the increase in TEAD-1 seemed to partly contribute to the activation of BNP promoter in the pBNP-tdTomato-positive cells.

### Effect of an extract of papillary tips on the blood pressure, serum BNP and urine cGMP concentration of SHR-SP rats

**Fig 7:**
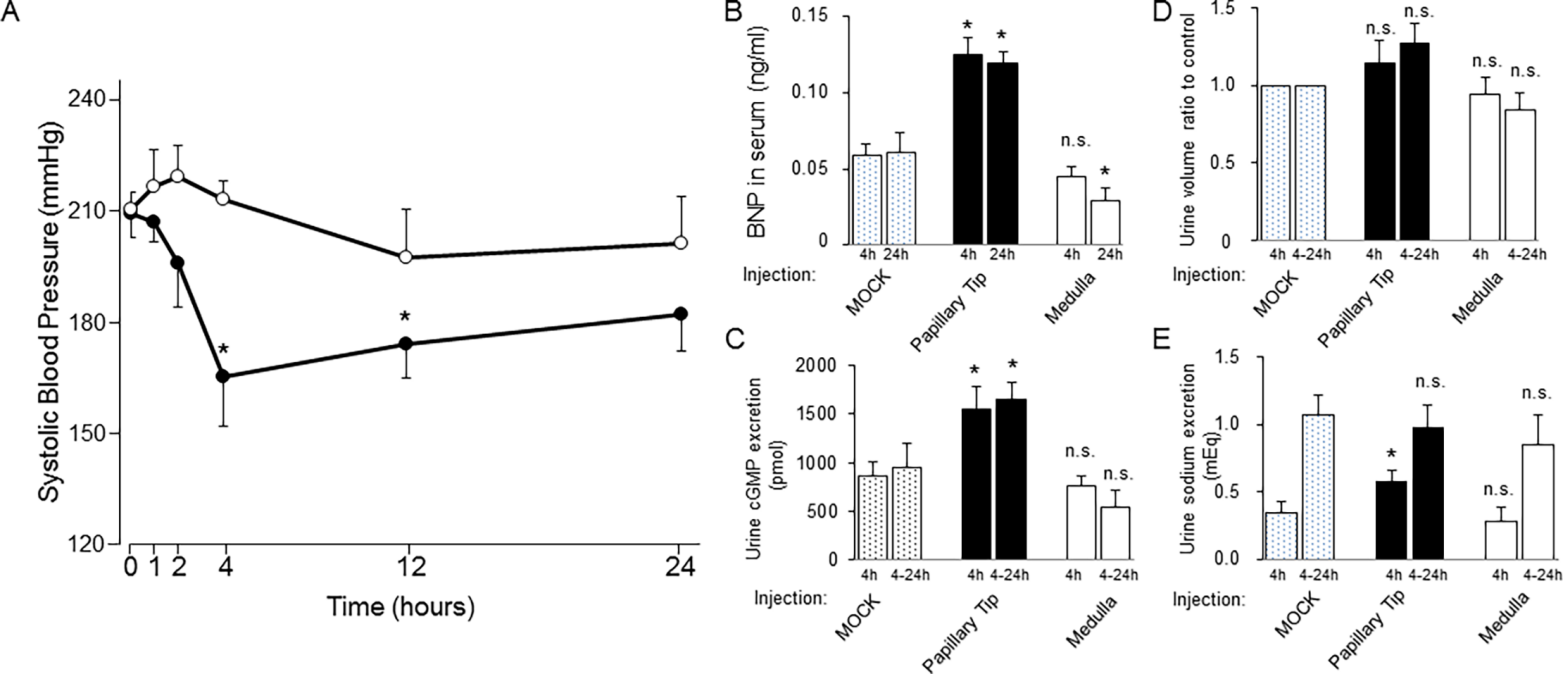
Effects of extracts of the papillary tip (●) and inner medulla (○) of the kidneys on stroke-prone spontaneously hypertensive rats (SHR-SP). A, Each extract was intraperitoneally administered to SHR-SP rats and their systolic blood pressure was followed for 24 h using the tail-cuff method. *P < 0.05 vs. basal blood pressure (at 0 h). n = 4 to 6. B, Measurement of serum BNP (n = 4 to 8) and C, cGMP in the urine 4h and 24 h after intraperitoneal injection of buffer alone or the extract of the papillary tip or the inner medulla. *P < 0.05 or n.s. = no significant difference vs. serum from SHR-SP rats treated with buffer alone (MOCK). n = 4 to 8. D, The ratio of urine volume to MOCK 4h and 24h after the injection. n.s. = no significant difference vs. the urine from SHR-SP rats treated with buffer alone (MOCK). n = 4 to 8. E, urine sodium excretion for 4h (0-4h) and 20h (4–24 h) after the injection of control buffer or extract of the papillary tip or the inner medulla into SHR-SP rats. n.s. = no significant difference vs. the urine from SHR-SP rats treated with buffer alone (MOCK). n = 4 to 8. For all figures, data indicate the mean +/- standard error of the mean.

It has been reported that the papillary medulla possesses the ability to reduce blood pressure via its vasodilatory activity [16, 17]. To measure this activity of the papillary medulla of transgenic mice, we injected an extract of the papillary tip intraperitoneally into SHR-SPs, an established hypertension model, and evaluated the changes in blood pressure and levels of serum BNP and its effector, urine cGMP. When compared to basal pressure, systolic blood pressure was significantly decreased 4 and 12 h after the extract of the papillary tip was injected intraperitoneally; in contrast, injection of an extract of the inner medulla had no effect (Fig 7A). The decrease in systolic blood pressure was accompanied by an increase in serum BNP 4h and 24h after the injection (Fig 7B) and urine cGMP excretion for the first 4h (0-4h) and the last 20h (4-24h) after the injection (Fig 7C). However, only a mild increase in urine volume (Fig 7D) and sodium excretion (Fig 7E) was observed, suggesting that the reduction in blood pressure was mainly due to a vasodilatory effect of the extract.

### Effect of an extract of papillary tips from rats with heart failure due to myocardial infarction on BNP expression in rat neonatal cardiomyocytes

**Fig 8:**
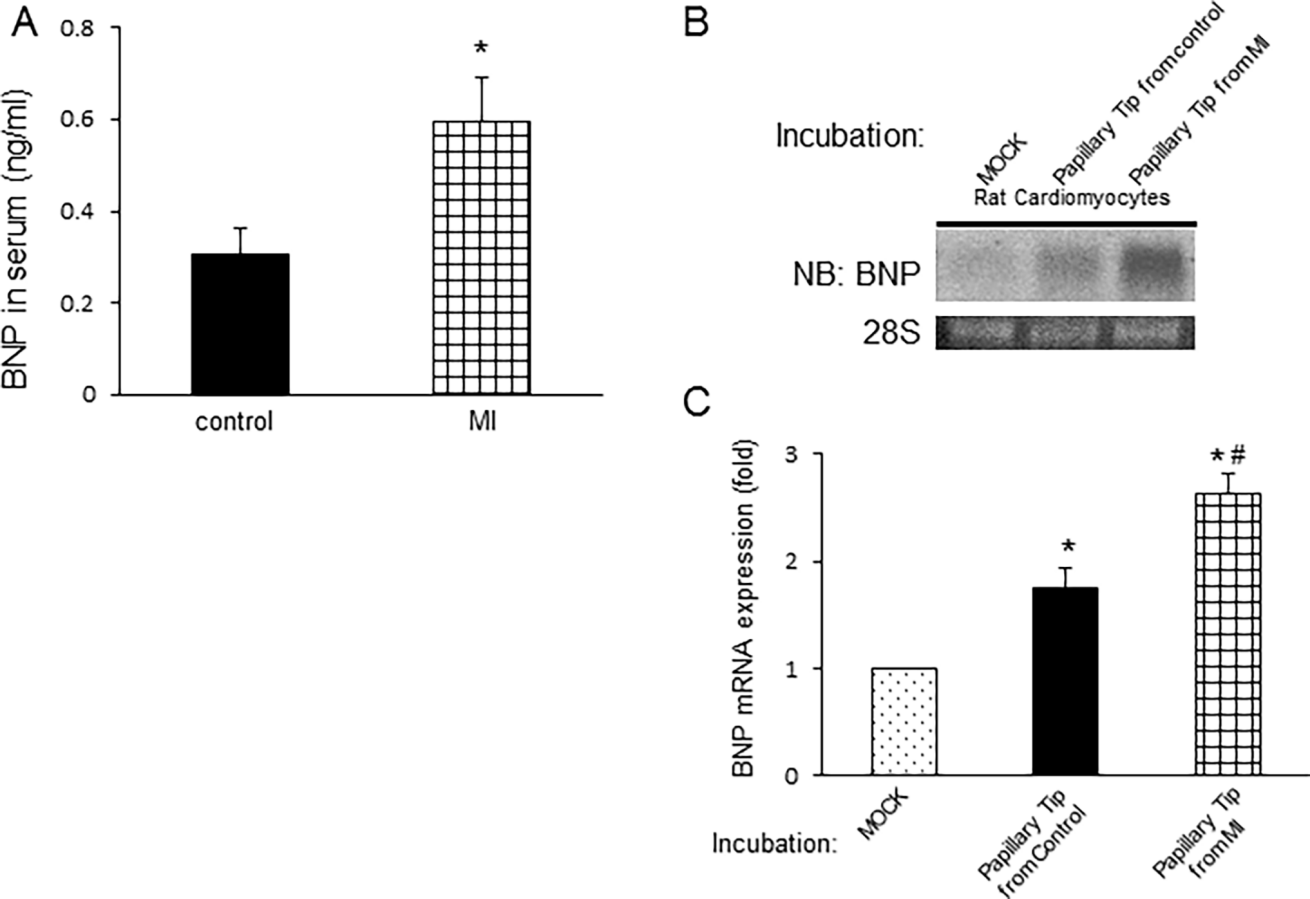
Effects of injection of extracts of the renal papillary tip from rats 5 days after ligation of the left anterior descending coronary artery on the expression of BNP in rat neonatal cardiomyocytes. A, Serum BNP was examined using a rat BNP enzyme-linked immunosorbent assay kit. n = 4. B, BNP mRNA expression was examined by Northern blot analysis in cardiomyocytes treated with control buffer or an extract of the renal papillary tip from control rats or from myocardial infarction (MI) model rats. *P < 0.05 vs. cardiomyocytes treated with the buffer alone (MOCK). #P < 0.05 vs. cardiomyocytes treated with the extract from control rat kidneys. n = 4. Data indicate the mean +/- standard error of the mean.

Since it is well-known that BNP levels are increased in patients with heart failure, we examined the effect of extracts of papillary tips from rats with heart failure on BNP induction. We studied myocardial infarction as a heart failure model. Ligation of the rats’ left anterior descending coronary artery was performed, and the effects of extracts from the papillary tips of kidneys of heart failure rats were examined five days after the induction of myocardial infarction. Serum BNP was increased in rats with myocardial infarction (Fig 8A). The induction of BNP was also increased in myocardial infarction as compared to control rats (Fig 8B and 8C), suggesting that BNP production induced by papillary tips partly caused continuous induction of BNP.

## Discussion

In this paper, using a transgenic BNP-promoter reporter-overexpressing mouse, we showed that this reporter was overexpressed specifically in the renal papilla. We further found that treatment with the renal papillary tip extract increased both the expression and secretion of BNP in primary cultured neonatal cardiomyocytes. Decrease in systolic blood pressure and increase in serum BNP and urine cGMP after intraperitoneal injection of the papillary tip extract were observed in a rat hypertensive model. We conclude from these data that the renal papillary tip plays an important role in the regulation of BNP.

BNP levels are known to be relatively higher in women, patients with chronic kidney disease and elderly persons with no heart disease [9]; however, the mechanism behind this increase remains unclear. In this study, by using several pBNP-tdTomato Tg mouse cell lines, including cells from elderly female mice, we found that activation of the BNP promoter was specific to the kidney papillary tip and was not accompanied by *BNP* mRNA expression.

Mouse 5’ flanking sequence (5’FS) upstream from the 5’ untranslated region is 77% and 90% homologous with human and rat BNP genes [8]. Mouse, human and rat proximal promoters contain a TATA box, GATA sites, a muscle-CAT binding site (MCAT) and an AP-1/CRE-like element. These sites are bound by the general transcriptional factor, transcription factor IID (TFIID), GATA-4, TEAD-1 and c-FOS (AP-1) [8, 18]. Indeed, the expression of BNP is regulated by GATA-4, TEAD-1 and c-FOS after stimulation by mechanical stretch and/or agonists [19–21]. We found that TEAD-1 was upregulated in pBNP-tdTomato-positive cells compared with tdTomato-negative cells (Fig 6D). These results suggest that the expression of tdTomato partly reflects the real transcriptional regulation of BNP, although BNP mRNA expression was not observed in pBNP-tdTomato-positive cells. This result was not due to the insertion sites of pBNP-tdTomato, because more than two independent lines of pBNP-tdTomato Tg mice showed tdTomato expression specifically in the renal papillary tip. Further, the exact reason why mRNA was not recognized in pBNP-tdTomato-positive cells in the papillary tips, and whether the promoter of BNP is partly activated by autocrine, paracrine or other mechanisms, remains unknown. It seems difficult to monitor the activation of BNP promoter correctly in vivo if we have developed transgenic mice with the use of 140kb 5’FS of BNP gene [22]. Hence, development of knock-in mice would be necessary for observation of the real promoter activity of BNP.

The pBNP-tdTomato-positive cells were interstitial cells and were not proliferative. Importantly, the renal papilla has been reported by several independent groups to express a depressor substance that acts as a counterpart to the renin-angiotensin-aldosterone system [16, 17]. This depressor substance is produced by renomedullary interstitial cells (RMICs). However, most researchers did not distinguish between the papillary tip and the entire inner medulla. Although the inclusions have not yet been fully elucidated, electron microscopy studies have shown that the contents consist of lipids, including free fatty acids and prostaglandins. However, the depressor agent does not appear to be a prostaglandin, a platelet-activating factor or nitric oxide [16, 23]. Interestingly, pBNP-tdTomato-positive cells were found in the papillary interstitial space and were cylindrical in shape, which is consistent with the localization and appearance of RMICs [24]. In the papillary tip, RMICs have abundant granules when the kidney is clipped, which degranulate after unclipping in a one-kidney, one-clip hypertensive rat model [24]. Thus, it remains unknown whether RMICs and the contents of the granules of RMICs are associated with the regulation of BNP. Additionally it is not clear which type of cells in the renal papilla can induce BNP production. Specific receptors for angiotensin II and natriuretic peptides have been found in tubular and interstitial cells [25, 26], although we observed no change in the expression of these peptides between the papillary tip and other portions of the kidneys.

Reportedly, a small change in basal BNP concentration contributes to a decrease in blood pressure and possibly to a decrease in cardiovascular events [7]. On the other hand, a large amount of BNP is secreted in patients with exacerbated heart failure. Thus, it is possible that blood pressure may respond to basal and stimulated secretion of BNP in different ways.

In this study, we observed an increase in the induction of BNP by the papillary tip of rats 5 days after the induction of myocardial infarction (Fig 8), suggesting that papillary tips may play important roles in regulating BNP expression in the acute myocardial infarction.

Further investigation is necessary to determine the relationship between the papillary depressor system and BNP regulation, and whether BNP production from the heart is partly regulated by the renal papillary tip in patients with cardiovascular diseases, such as heart failure, hypertension and chronic kidney disease.

## Supporting information

S1 Fig In situ hybridization of BNP. (A) in wild-type mouse heart three days after induction of myocardial infarction by left descending coronary artery ligation, and (B) in renal papillary tips from adult Tg mice. n = 3.
(PPTX)

S2 Fig Isolation of pBNP-tdTomato-positive and -negative cells by fluorescence activated cell sorting (FACS) from renal papillary tips of Tg mice. The P4 and P2 areas have been defined as tdTomato-positive and -negative cells respectively. n = 3.
(PPTX)

S1 File Supplemental materials and methods. (DOCX)
