# Supplementary material for: Renal papillary tip extract stimulates BNP production and excretion from cardiomyocytes
Source: PLoS One. 2018 May 7;13(5):e0197078. doi: 10.1371/journal.pone.0197078 (PMC5937764; doi:10.1371/journal.pone.0197078)
Supplement: S1 Fig — (A) in wild-type mouse heart three days after induction of myocardial infarction by left descending coronary artery ligation, and (B) in renal papillary tips from adult Tg mice. n = 3. (PPTX) [file pone.0197078.s001.pptx]

## Slide 1
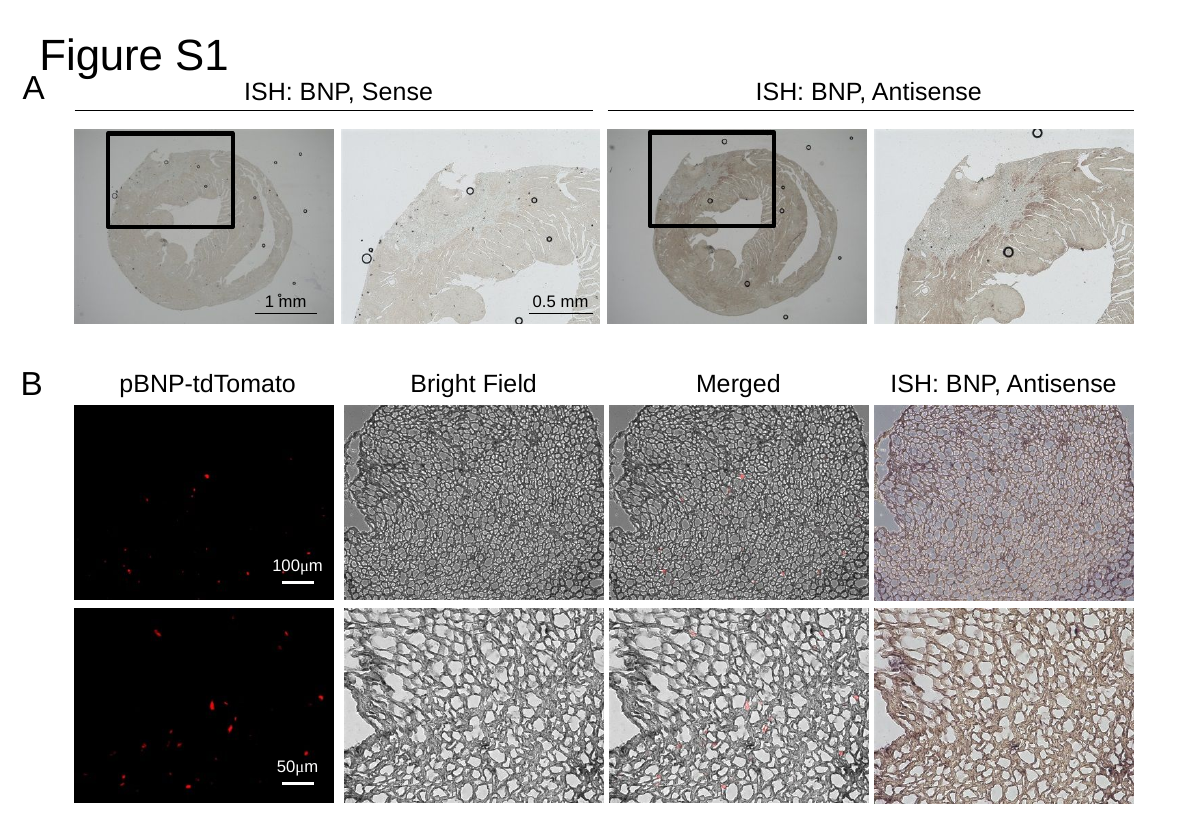

Figure S1
A
ISH: BNP, Sense
ISH: BNP, Antisense
1 mm
0.5 mm
B
pBNP-tdTomato
Bright Field
Merged
ISH: BNP, Antisense
100μm
50μm
