# Supplementary material for: Renal papillary tip extract stimulates BNP production and excretion from cardiomyocytes
Source: PLoS One. 2018 May 7;13(5):e0197078. doi: 10.1371/journal.pone.0197078 (PMC5937764; doi:10.1371/journal.pone.0197078)
Supplement: S2 Fig — The P4 and P2 areas have been defined as tdTomato-positive and -negative cells respectively. n = 3. (PPTX) [file pone.0197078.s002.pptx]

## Slide 1
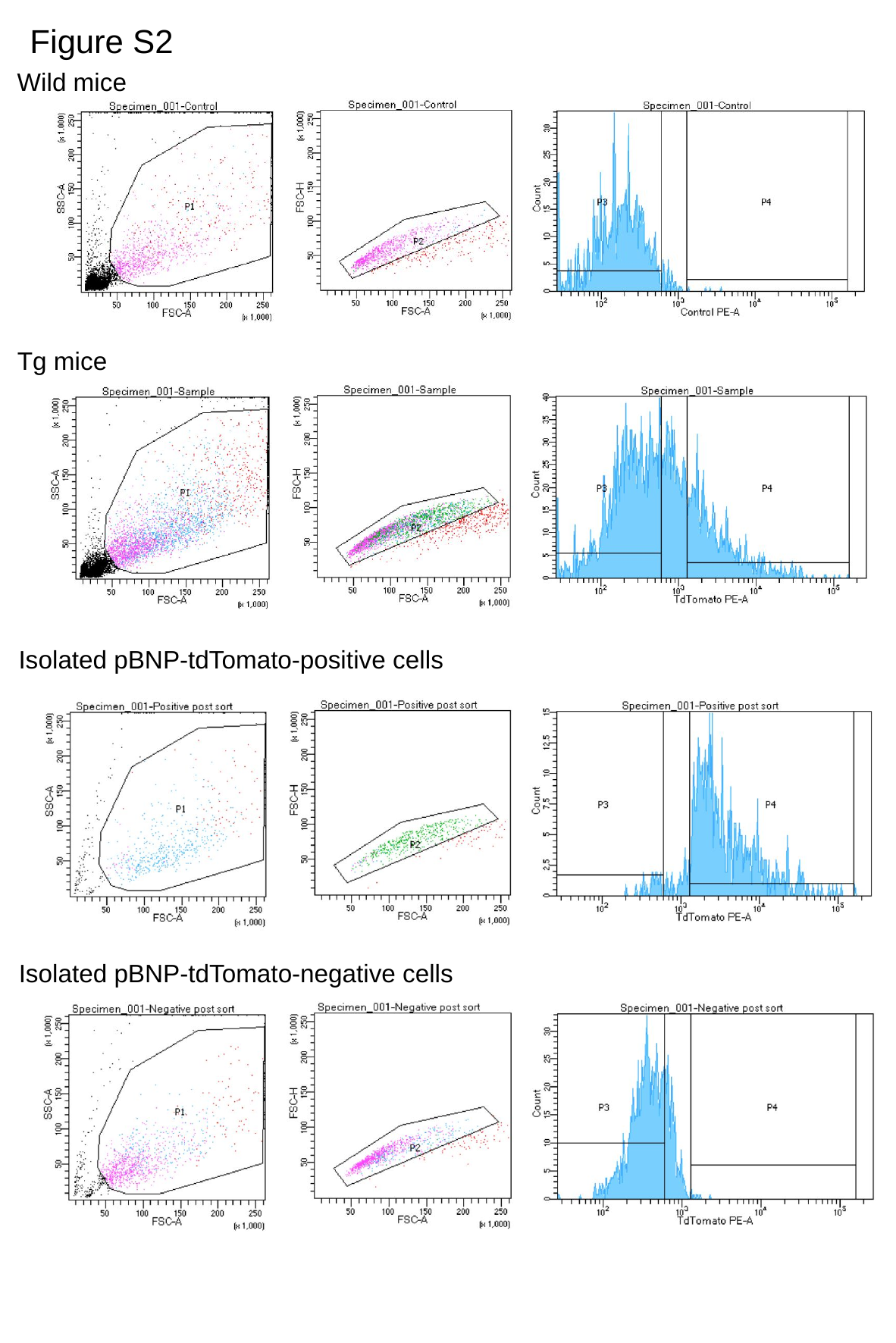

Figure S2
Wild mice
Tg mice
Isolated pBNP-tdTomato-positive cells
Isolated pBNP-tdTomato-negative cells
