# Supplementary material for: Renal papillary tip extract stimulates BNP production and excretion from cardiomyocytes
Source: PLoS One. 2018 May 7;13(5):e0197078. doi: 10.1371/journal.pone.0197078 (PMC5937764; doi:10.1371/journal.pone.0197078)
Supplement: S1 File — (DOCX) [file pone.0197078.s003.docx]

**Supplemental materials and methods**

**In situ hybridization of BNP in mouse kidneys and heart**

Non-radioactive in situ hybridization in sections was performed as described previously [[1](#_ENREF_1)]. Kidneys and heart were fixed in 4% paraformaldehyde, embedded in Tissue-Tek OCT compound (Sakura Finetek, Tokyo, Japan) and snap-frozen in liquid nitrogen; 8 μm cryosections were used. Mouse NPPB cDNA (ATG to stop) was amplified from mouse heart cDNA and subcloned into the pBluescript SK vector. The fragment was sequenced to confirm that the PCR-amplified cDNA was identical to the original predicted sequence. The cDNA was linearized with *Not*I and labeled with T7 RNA for the antisense probe or linearized with *Sal*I and labeled with T3 for the sense probe.

**References**

[1] Yoshida T, Akatsuka T, Imanaka-Yoshida K. Tenascin-C and integrins in cancer. Cell Adh Migr. 2015;9:96-104.
